# The systemic immune-inflammation index as a superior predictor of functional outcome following mechanical thrombectomy for acute ischemic stroke: a retrospective cohort study

**DOI:** 10.3389/fneur.2026.1757013

**Published:** 2026-02-25

**Authors:** Bo Zhou, Yu Liu, Menglu Zhang, Qingtao Xie, Shiqin Ju, Qingqing Liu, Yu Feng, Yanbo Cheng

**Affiliations:** 1 Department of Neurology, The Affiliated Hospital of Xuzhou Medical University, Xuzhou, China; 2 Xuzhou Medical University, Xuzhou, China; 3 Department of Geriatrics, Xuzhou Cancer Hospital, Xuzhou, China

**Keywords:** acute ischemic stroke, mechanical thrombectomy, neutrophil lymphocyte ratio, platelet lymphocyte ratio, systemic immune-inflammation index

## Abstract

**Objective:**

Despite high recanalization rates with mechanical thrombectomy (MT) for acute ischemic stroke (AIS), functional outcomes remain variable. Systemic inflammation is a key driver of secondary brain injury post-reperfusion. The systemic immune-inflammation index (SII), calculated as (platelet count × neutrophil count)/lymphocyte count, integrates multiple inflammatory pathways and has shown prognostic value in cardiovascular diseases and stroke treated with intravenous thrombolysis. However, its role in predicting outcomes specifically for AIS patients undergoing MT remains underexplored. This study aimed to develop and validate an SII-based model for predicting 90-day functional outcomes after MT and to compare its performance with traditional inflammatory biomarkers, namely neutrophil-to-lymphocyte ratio (NLR) and platelet-to-lymphocyte ratio (PLR).

**Methods:**

We retrospectively analyzed data from 387 AIS patients treated with MT. The cohort had a median age of 68 years [interquartile range (IQR): 59–75], 67.2% were male, and the median time from stroke onset to thrombectomy was 340 min (IQR: 242.5–465.5). Inflammatory markers were measured at admission, such as SII, platelet lymphocyte ratio (PLR), neutrophil lymphocyte ratio (NLR) and 90-day modified Rankin Scale (mRS) scores. Patients were divided into good (90-day mRS ≤ 2; *n* = 151) and poor (mRS > 2; *n* = 236) outcome groups. We constructed and compared four logistic regression models: clinical baseline, baseline + SII, baseline + PLR, and baseline + NLR. Model performance was assessed using the area under the receiver operating characteristic curve (AUC), net reclassification improvement (NRI), integrated discrimination improvement (IDI), calibration, and decision curve analysis (DCA).

**Results:**

SII alone showed higher predictive accuracy (AUC: 0.834) than PLR or NLR. The optimal model (baseline + SII) achieved an AUC of 0.863, significantly improving outcome prediction over the baseline model (AUC: 0.655). Shapley Additive exPlanations (SHAP) analysis confirmed SII as the most influential variable (74.2% contribution). The model demonstrated good calibration and clinical utility across a range of probability thresholds.

**Conclusion:**

A model incorporating the SII provides superior accuracy for predicting 90-day functional outcome after MT compared to models using NLR or PLR. As an easily obtainable composite biomarker, SII enhances risk stratification and could aid early clinical decision-making for AIS patients undergoing endovascular therapy.

## 1 Introduction

Acute ischemic stroke (AIS) is one of the leading causes of death and disability worldwide (1). Currently, mechanical thrombectomy (MT) has been established as one of the most effective treatments for AIS patients with large vessel occlusion (2). However, despite successful recanalization achieved by MT, functional outcomes remain highly variable, with approximately 50% of patients experiencing poor functional outcome at 90 days (3). Accurate prediction of functional outcome is therefore crucial for risk stratification, individualized management, and optimization of post-procedural care.

Increasing evidence highlights the critical role of systemic inflammation in the pathophysiology of AIS and its impact on outcome after reperfusion therapy (4). Ischemic stroke triggers a robust inflammatory cascade that extends beyond the brain, involving peripheral immune activation and persistent whole-brain inflammation that can exacerbate secondary injury and impair neurological recovery (4).

In recent years, the systemic immune-inflammation index (SII), calculated as (platelet count × neutrophil count)/lymphocyte count, has emerged as a comprehensive inflammatory marker integrating pathways of thrombosis, innate immunity, and adaptive immune suppression. SII has shown prognostic value in cardiovascular diseases (5, 6) and various malignancies (7, 8). In the context of AIS, elevated admission SII has been associated with early neurological deterioration and unfavorable 3-month outcomes in patients treated with intravenous thrombolysis (9), symptomatic intracranial hemorrhage after endovascular treatment (10), and higher risk of poor functional outcome (11, 12). These findings suggest that SII may capture multidimensional inflammatory and immune dysregulation more effectively than traditional markers such as the neutrophil-to-lymphocyte ratio (NLR) or platelet-to-lymphocyte ratio (PLR) (13).

Given the persistent inflammatory burden following reperfusion and the established associations between SII and adverse events in AIS, incorporating SII into prognostic models may improve risk prediction after MT. The present study aimed to develop and validate an interpretable predictive modeling framework based on SII for 90-day functional outcome following MT in AIS patients. We further compared the predictive performance of SII with that of NLR and PLR to determine the optimal inflammatory marker for integration into clinical prognostic models.

## 2 Information and methods

### 2.1 Study population

We retrospectively collected Patients with AIS between January 2022 and December 2024 at the Affiliated Hospital of Xuzhou Medical University. The study was conducted in accordance with the Declaration of Helsinki and approved by the Ethics Committee of the Affiliated Hospital of Xuzhou Medical University. Inclusion criteria:(1) diagnosed with AIS according to World Health Organization criteria with complete baseline and follow-up data; (2) confirmed large vessel occlusion by imaging and treated with MT; and (3) time from onset to start of thrombolysis was <6 h, which could be extended to 16 h or 24 h by meeting the inclusion criteria for the DEFUSE 3, DAWN, or BAOCHE studies (14–16); (4) Age > 18 years. Exclusion criteria: (1) CT of the head indicates intracranial hemorrhage or other conditions (e.g., intracranial aneurysm) associated with high bleeding risk that would contraindicate thrombectomy; (2) severe allergy to contrast media; (3) pregnant and breastfeeding females; (4) patients with serious diseases, such as renal failure, severe hepatic insufficiency, or cancer; (5) patients with autoimmune diseases; (6) patients with immunosuppressive drugs or antibiotic use; (5) 90d postoperative loss of visit.

### 2.2 Data collection

We collected patients’ general information and medical history (including age, sex, diabetes mellitus, hypertension, and atrial fibrillation), preoperative National Institutes of Health Stroke Scale (NIHSS) scores, modified Rankin Scale (mRS) scores, and 90-day postoperative mRS scores. Stroke characteristics [Trial of Org 10,172 in Acute Stroke Treatment (TOAST) classification and site of onset] and procedural details (onset-to-puncture time, procedure duration, number of passes, thrombectomy method, pre-procedure intravenous thrombolysis, stent use, and the grade of revascularization after the procedure [assessed by the Thrombolysis In Cerebral Infarction (TICI) grading]) were recorded. The TICI grading is used to assess the degree of cerebrovascular recanalization, primarily evaluating post-procedural blood flow restoration following endovascular treatment for AIS. Laboratory indices (platelet, neutrophil, and lymphocyte counts) were collected from peripheral blood samples obtained immediately upon the patient’s admission, prior to any endovascular intervention. The SII was calculated as (platelet count × neutrophil count)/lymphocyte count, and we also calculated the PLR which was calculated as platelet count/lymphocyte count and NLR which was calculated as neutrophil count/lymphocyte count.

### 2.3 Follow-up and grouping

Patients or their family members were followed up by telephone or outpatient clinic at 90d postoperatively, and the functional outcome was assessed according to the mRS score. mRS ≤ 2 points were included in the good functional outcome group, and mRS > 2 points were included in the bad functional outcome group, and the data of the two groups were statistically analyzed to explore the effects of NLR, PLR, and SII levels on the patients’ poor functional outcome at 90d postoperatively after MT.

### 2.4 Statistical methods

All the measured data were statistically described by mean ± standard deviation to satisfy normality, and the *t*-test was used for the differences between the two groups; non-normal distribution was statistically described by median (percentile), and the Wilcoxon’s rank sum test was used for the differences between the two groups. The statistical description of the count data was based on the number of cases (%), and the differences between groups were tested by *X*^2^ or Fisher’s exact probability test. The Wilcoxon rank sum test was used for differences between the two groups for grade information. Based on the results of intergroup variability, in order to study the analysis of risk factors related to the functional outcome of AIS patients after MT, a one-way logistic regression model was used to analyze the statistically significant differences between good and poor functional outcome of AIS patients after MT, and based on these results, we identified key clinical predictors and constructed logistic regression models. The baseline clinical model included onset-to-puncture time, procedure duration, and admission NIHSS. We then built three additional models by adding each inflammatory index to the baseline variables: Model 2 = baseline + SII, Model 3 = baseline + PLR, and Model 4 = baseline + NLR. Model performance was evaluated by ROC curves, and the optimal model was selected using NRI and IDI analyses, as well as calibration curves and DCA. The area under the ROC curve (AUC) was used to predict the performance of the models, and the optimal prediction model was constructed by using the NRI and IDI analyses, and the area under the ROC curve (AUC) was used to evaluate the value of the different prediction models and the performance of the models for predicting the clinical outcomes of patients with AIS after mechanical thrombus extraction. The calibration curves and the Hosmer–Lemeshow test were used to evaluate model fit, while internal validation was performed using bootstrap resampling with 1,000 repetitions to ensure the robustness of the primary model. Model fit was assessed using calibration curves and the Hosmer–Lemeshow test. To evaluate the internal validity and correct for potential overfitting, internal validation was performed using bootstrap resampling with 1,000 repetitions. This generated an optimism-corrected estimate of model performance (e.g., AUC), providing a more realistic assessment of the model’s generalizability within the study cohort. Finally, decision curve analysis (DCA) was employed to assess the clinical value of the models. To further assess the predictive efficacy of SII, PLR and NLR on the functional outcome of patients with AIS after MT, ROC curves were analyzed using unadjusted analysis and multivariable-adjusted ROC analysis to account for potential confounders. *p* < 0.05 was considered statistically significant. R4.3.2 software was used for basic statistical analysis, and Python3.9 software was used for SHapley Additive exPlanations (SHAP) visualization.

## 3 Outcomes

### 3.1 Overall results

Based on the inclusion and exclusion criteria, 387 cases were extracted from hospital information system (HIS) database of AIS in our hospital, out of which 236 cases occurred with poor functional outcome and the incidence of poor functional outcome was 60.98%. Table 1 compares baseline characteristics of the two outcome groups. Compared to patients with good functional outcome, those with poor functional outcome had significantly longer onset-to-puncture and procedure durations, higher NIHSS scores at reperfusion, and higher SII, PLR, and NLR values (all *p* < 0.05).

**Table 1:** Comparison of clinical baseline data between the two groups.

Variable	Good functional outcome (*N* = 151)	Poor functional outcome (*N* = 236)	Total (*N* = 387)	*t*-test/*X*^2^	*p*-value
Gender				0.015	0.902
Female *n*%	49 (32.45%)	78 (33.05%)	127 (32.82%)		
Male *n*%	102 (67.55%)	158 (66.95%)	260 (67.18%)		
Age median (years)	67.00 [58.00; 73.00]	69.00 [59.00; 77.00]	68.00 [59.00; 75.00]	−1.902	0.057
TOAST					
1 = ACI; 2 = CCI				0.988	0.320
1	107 (70.86%)	178 (75.42%)	285 (73.64%)		
2	44 (29.14%)	58 (24.58%)	102 (26.36%)		
Affected area					
1 = AC; 2 = PC;				1.490	0.222
1	126 (83.44%)	185 (78.39%)	311 (80.36%)		
2	25 (16.56%)	51 (21.61%)	76 (19.64%)		
MT times				4.173	0.243
1	115 (76.16%)	160 (67.80%)	275 (71.06%)		
2	21 (13.91%)	51 (21.61%)	72 (18.60%)		
3	13 (8.61%)	23 (9.75%)	36 (9.30%)		
4	2 (1.32%)	2 (0.85%)	4 (1.03%)		
MT techniques					
1 = stent retriever 2 = aspiration 3 = combined thrombectomy 4 = arterial thrombolysis				6.320	0.097
1	128 (84.77%)	179 (75.85%)	307 (79.33%)		
2	8 (5.30%)	25 (10.59%)	33 (8.53%)		
3	15 (9.93%)	29 (12.29%)	44 (11.37%)		
4	0 (0.0%)	3 (1.27%)	3 (0.78%)		
Intravenous thrombolysis (yes = 1; no = 0)				0.082	0.774
0	88 (58.28%)	141 (59.75%)	229 (59.17%)		
1	63 (41.72%)	95 (40.25%)	158 (40.83%)		
Onset to puncture time/min	300.00 [220.00; 432.5]	365.00 [272.50; 476.5]	340.00 [242.50; 465.5]	−3.166	0.002
Operating time/min	90.00 [60.00; 120.00]	100.00 [80.00; 140.00]	97.00 [70.00; 125.00]	−3.351	0.001
Hypertension (yes = 1; no = 0)				1.576	0.209
0	86 (56.95%)	119 (50.42%)	205 (52.97%)		
1	65 (43.05%)	117 (49.58%)	182 (47.03%)		
Diabetes (yes = 1; no = 0)				0.936	0.333
0	128 (84.77%)	191 (80.93%)	319 (82.43%)		
1	23 (15.23%)	45 (19.07%)	68 (17.57%)		
Atrial fibrillation (yes = 1; no = 0)				1.302	0.254
0	108 (71.52%)	181 (76.69%)	289 (74.68%)		
1	43 (28.48%)	55 (23.31%)	98 (25.32%)		
TICI grading				5.388	0.145
0	3 (1.99%)	2 (0.85%)	5 (1.29%)		
1	1 (0.66%)	9 (3.81%)	10 (2.58%)		
2	16 (10.60%)	32 (13.56%)	48 (12.40%)		
3	131 (86.75%)	193 (81.78%)	324 (83.72%)		
NIHSS 0	16.00 [11.00; 25.00]	20.00 [14.00; 30.00]	19.00 [12.00; 27.00]	−3.425	0.001
mRS 0				3.146	0.207
3	3 (1.99%)	5 (2.12%)	8 (2.07%)		
4	68 (45.03%)	85 (36.02%)	153 (39.53%)		
5	80 (52.98%)	146 (61.86%)	226 (58.40%)		
SII	566.74 [383.78; 891.60]	1407.66 [965.41; 2358.12]	1012.23 [604.65; 1748.30]	−11.533	0.000
PLR	110.74 [79.24; 155.11]	198.18 [137.25; 283.33]	157.27 [108.50; 235.83]	−10.169	0.000
NLR	3.08 [2.31; 4.58]	7.34 [4.68; 11.21]	5.36 [3.13; 8.68]	−11.028	0.000

TOAST, Trial of Org 10,172 in Acute Stroke Treatment classification system; ACI, arterial-derived cerebral infarction; CCI, cardiac-derived cerebral infarction; AC, anterior circulation; PC, posterior circulation; TICI, thrombolysis in Cerebral Infarction Classification; NIHSS 0, National Institutes of Health Stroke Scale score at the time of admission; mRS0, 0 Modified Rankin Scale score at the time of admission; SII, systemic immune-inflammation index; PLR, platelet-to-lymphocyte ratio; NLR, neutrophil-to-lymphocyte ratio.

### 3.2 Research on the value of comprehensive inflammation indexes in predicting clinical outcomes

**Figure 1:**
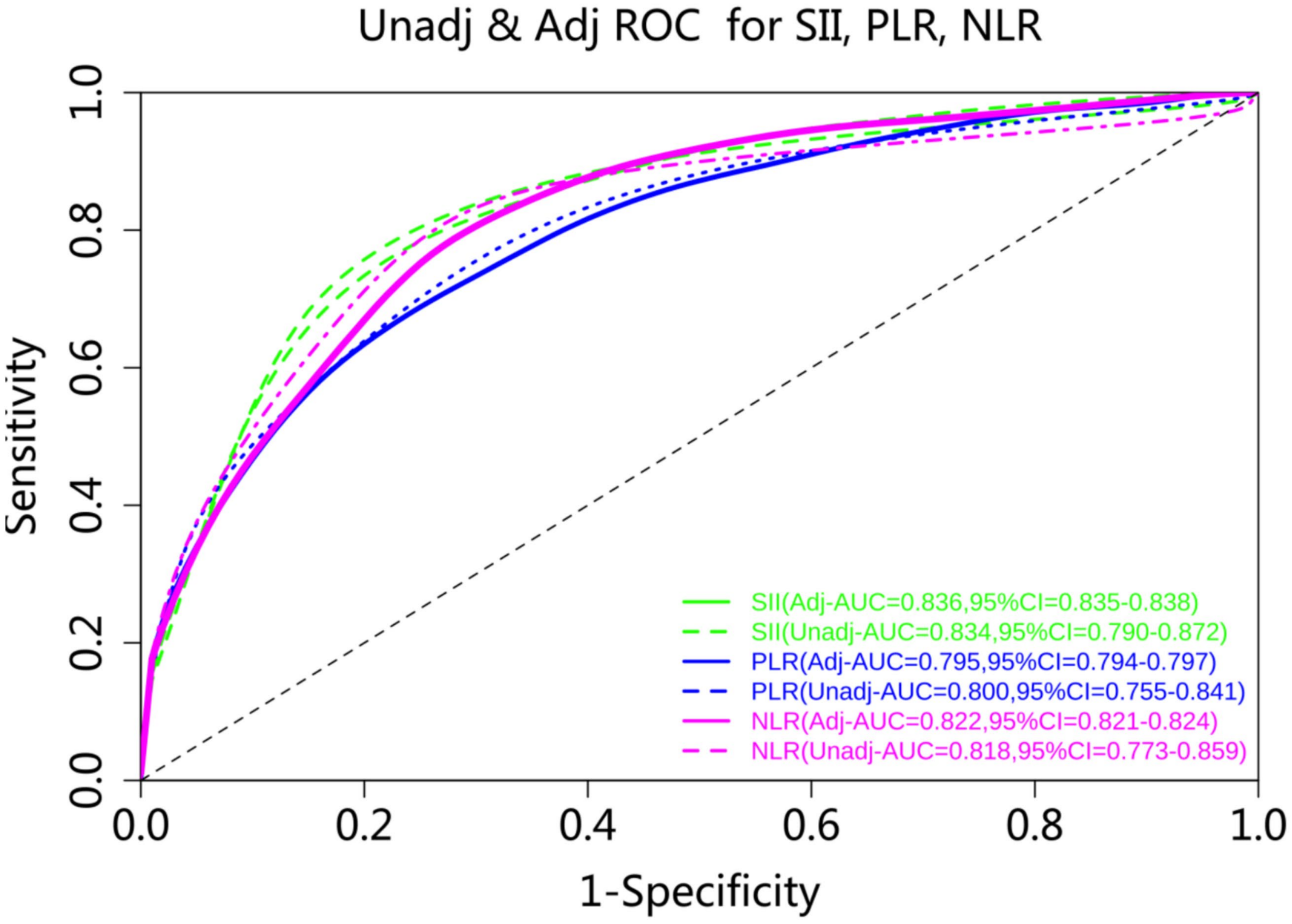
ROC curve plot of comprehensive inflammatory indicators predicting clinical outcomes.

Since the present study found statistically significant differences in the distribution of demographic and clinical indexes such as onset-to-puncture time (min), operating time (min), and NIHSS before MT between the two groups, in order to better assess the effectiveness of SII, PLR, and NLR in predicting clinical outcomes, the present study plotted the ROC curves of multivariable-adjusted ROC analysis and uncorrected covariates to illustrate in depth the predictive value of each comprehensive inflammation index. As shown in Figure 1, SIl had the greatest predictive efficacy, with an AUC = 0.834 (95% CI = 0.790–0.874) for uncorrected confounders and 0.841 (95% CI = 0.841–0.844) for multivariable-adjusted ROC analysis NLR was the next most effective predictor with an AUC = 0.820 (95% CI = 0.774–0.859) for uncorrected covariates; and NLR, with an AUC = 0.820 (95% CI = 0.774–0.859) for uncorrected covariates, and after correcting for other influences its AUC = 0.818 (95% CI = 0.818–0.821); and finally PLR, with an AUC = 0.800 (95% CI = 0.754–0.843) uncorrected for confounders, and after correcting for other influences its AUC = 0.810 (95% C1 = 0.809–0.812) (see Figure 1). The area under the ROC curve based on the above indicators was greater than 0.7, proving that the indicators SII, PLR, and NLR all have some predictive value.

### 3.3 Analysis and modeling of factors affecting functional outcome

**Figure 2:**
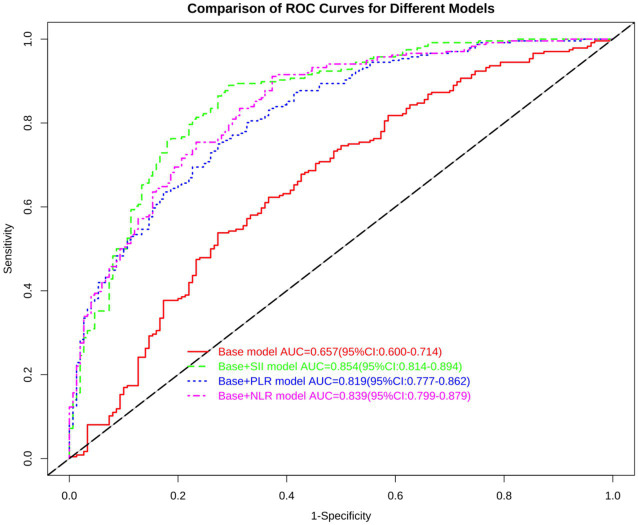
ROC curve analysis of different clinical prediction models.

We performed multivariate logistic regression with poor functional outcome as the dependent variable. The baseline model (Model 1) included the key clinical predictors (onset-to-puncture time, procedure time, and NIHSS at reperfusion). We then built three additional models by adding each inflammatory marker: Model 2 = baseline + SII, Model 3 = baseline + PLR, and Model 4 = baseline + NLR. As shown in Tables 2–4 and Figure 2, the model including SII (Model 2) showed the best predictive performance. Although our primary model is based on logistic regression—a standard and highly interpretable method for clinical risk prediction—its interpretability was further enhanced by employing SHAP analysis. This approach allowed us to move beyond traditional regression coefficients, providing a detailed visualization of how each predictor contributes to individual patient risk estimates.

**Table 2:** Results of AUC, sensitivity, specificity and other evaluation indices of the ROC for different models.

Variable	Cutoff	AUC (95% CI)	*p*-value	ACC	Sensitivity	Specificity	PPV	NPV
Model 1	0.113	0.655 (0.597, 0.712)	<0.001	0.669	0.826	0.424	0.691	0.610
Model 2	0.497	0.863 (0.825, 0.901)	<0.001	0.806	0.839	0.755	0.843	0.750
Model 3	0.017	0.830 (0.789, 0.871)	<0.001	0.762	0.809	0.689	0.803	0.698
Model 4	0.084	0.844 (0.804, 0.883)	<0.001	0.775	0.771	0.781	0.847	0.686

ACC, accuracy; PPV, positive predictive value; NPV, negative predictive value.

**Table 3:** ROC two-by-two comparison results for different models.

Comparative Model 1	Comparative Model 2	Statistic *Z*	*p*-value
Model 1	Model 2	−7.208	<0.0001
Model 1	Model 3	−6.075	<0.0001
Model 1	Model 4	−6.515	<0.0001
Model 2	Model 3	2.520	0.012
Model 2	Model 4	1.813	0.070
Model 3	Model 4	−0.896	0.370

**Table 4:** Results of two-by-two comparison of the performance of different models.

Comparison	NRI (categorical) [95% CI]	NRI (continuous) [95% CI]	IDI [95% CI]
Model 1 vs. Model 2	0.5349 [0.4497–0.6201]; *p* < 0.001	1.0778 [0.9153–1.2403]; *p* < 0.001	0.2901 [0.2483–0.3318]; *p* < 0.001
Model 1 vs. Model 3	0.4167 [0.3243–0.5092]; *p* < 0.001	0.8585 [0.6776–1.0393]; *p* < 0.001	0.2477 [0.2059–0.2894]; *p* < 0.001
Model 1 vs. Model 4	0.4443 [0.3543–0.5343]; *p* < 0.001	0.9284 [0.7523–1.1045]; *p* < 0.001	0.2654 [0.2235–0.3072]; *p* < 0.001
Model 2 vs. Model 3	−0.1356 [−0.2362 to −0.0351]; *p* = 0.008	−0.3398 [−0.541 to −0.1386]; *p* = 0.001	−0.0424 [−0.0704 to −0.0144]; *p* = 0.003
Model 2 vs. Model 4	−0.1568 [−0.2383 to −0.0753]; *p* < 0.001	−0.2662 [−0.468 to −0.0643]; *p* = 0.010	−0.0247 [−0.0484 to 0.001]; *p* = 0.041
Model 3 vs. Model 4	−0.0212 [−0.1238 to 0.0815]; *p* = 0.686	0.2699 [0.0675–0.4723]; *p* = 0.009	0.0177 [−0.0171–0.0524]; *p* = 0.319

After the Delong test, it was found that the difference in the area of the ROC curve of Model 2 was statistically significant (*p* < 0.05) compared with that of Model 1 and Model 3, and the difference was not statistically significant (*p* > 0.05) compared with that of Model 4, and the difference in the area of the ROC curve of Model 2 was statistically significant (*p* > 0.05). However, after NRI and IDI analyses, it was found that Model 4, when compared with Model 2, had an NRI (categorical) of −0.1568 [(95% CI: −0.2383 to −0.0753); *p* < 0.001]; an NRI (continuous) of −0.2662 [(95% CI: −0.468 to 0.0643); *p* = 0.010]; an IDI of −0.0247 [(95% CI: −0.0484 to −0.001); *p* = 0.041; and an IDI of −0.041. 0.0643; *p* = 0.010]; IDI was −0.0247 [(95% CI: −0.0484 to −0.001); *p* = 0.041], which shows that Model 2 has better comprehensive performance than Model 4 and is the optimal prediction model. The result is the formula for the optimal prediction equation: prognostic bad = −3.576 + 0.001 * OnsettoPunctureTime + 0.007 * procedure time + 0.031 * nihss0 + 0.002 * SII.

And, OnsettoPunctureTime, procedure time, nihss0 and SII were prognostic risk factors (OR all >1, *p* < 0.05). For details, see Table 5.

**Table 5:** Results of unifactorial and multifactorial logistic regression analyses influencing prognosis

Variables	Single factor logistic		Model 1		Model 2		Model 3		Model 4	
	OR (95% CI)	*p*-value	OR (95% CI)	*p*-value	OR (95% CI)	*p*-value	OR (95% CI)	*p*-value	OR (95% CI)	*p*-value
OTPT	1.001 (1.000, 1.002)	0.030	1.001 (1.000, 1.002)	0.039	1.001 (1.000, 1.002)	0.046	1.001 (1.000, 1.003)	0.041	1.001 (1.000, 1.002)	0.051
Procedure time	1.006 (1.002, 1.011)	0.007	1.005 (1.001, 1.010)	0.028	1.007 (1.001, 1.012)	0.017	1.007 (1.002, 1.013)	0.011	1.006 (1.001, 1.011)	0.024
NIHSS0	1.039 (1.015, 1,064)	0.001	1.041 (1.016, 1.066)	0.001	1.031 (1.003, 1.060)	0.028	1.041 (1.013, 1.070)	0.004	1.025 (0.997, 1.053)	0.082
SII	1.002 (1.002, 1.003)	0.000			1.002 (1.002, 1.003)	0.000				
PLR	1.016 (1.012, 1.020)	0.000					1.017 (1.013, 1.021)	0.000		
NLR	1.505 (1.370, 1.672)	0.000							1.513 (1.366, 1.677)	0.000

OTPT, onset-to-puncture time; NIHSS 0, National Institutes of Health Stroke Scale score at the time of admission; SII, systemic immune-inflammation index; PLR, platelet-to-lymphocyte ratio; NLR, neutrophil-to-lymphocyte ratio.

### 3.4 Visual construction and evaluation of the optimal prediction model

**Figure 3:**
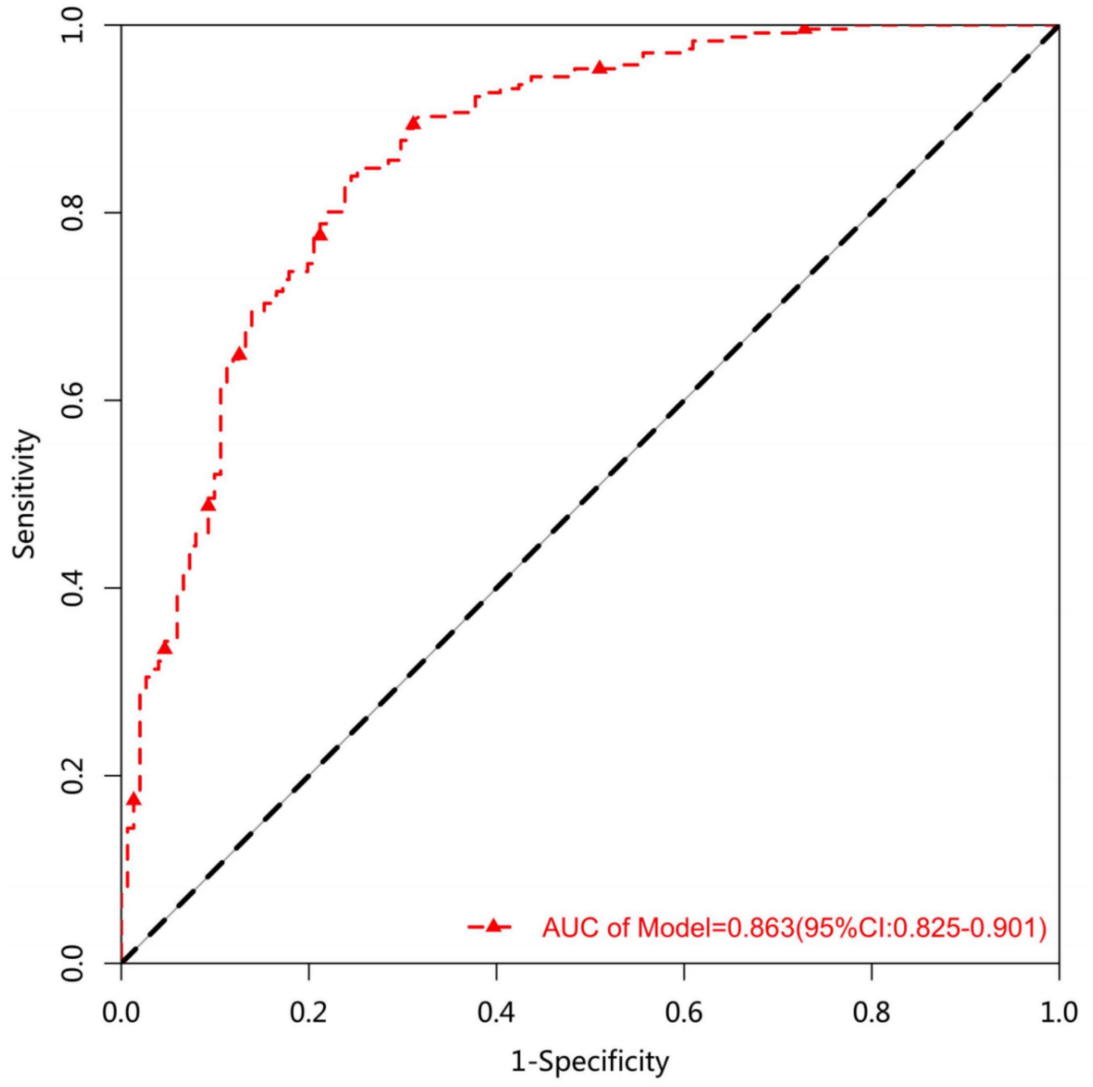
Predictive model effectiveness evaluation chart ROC curve.

**Figure 4:**
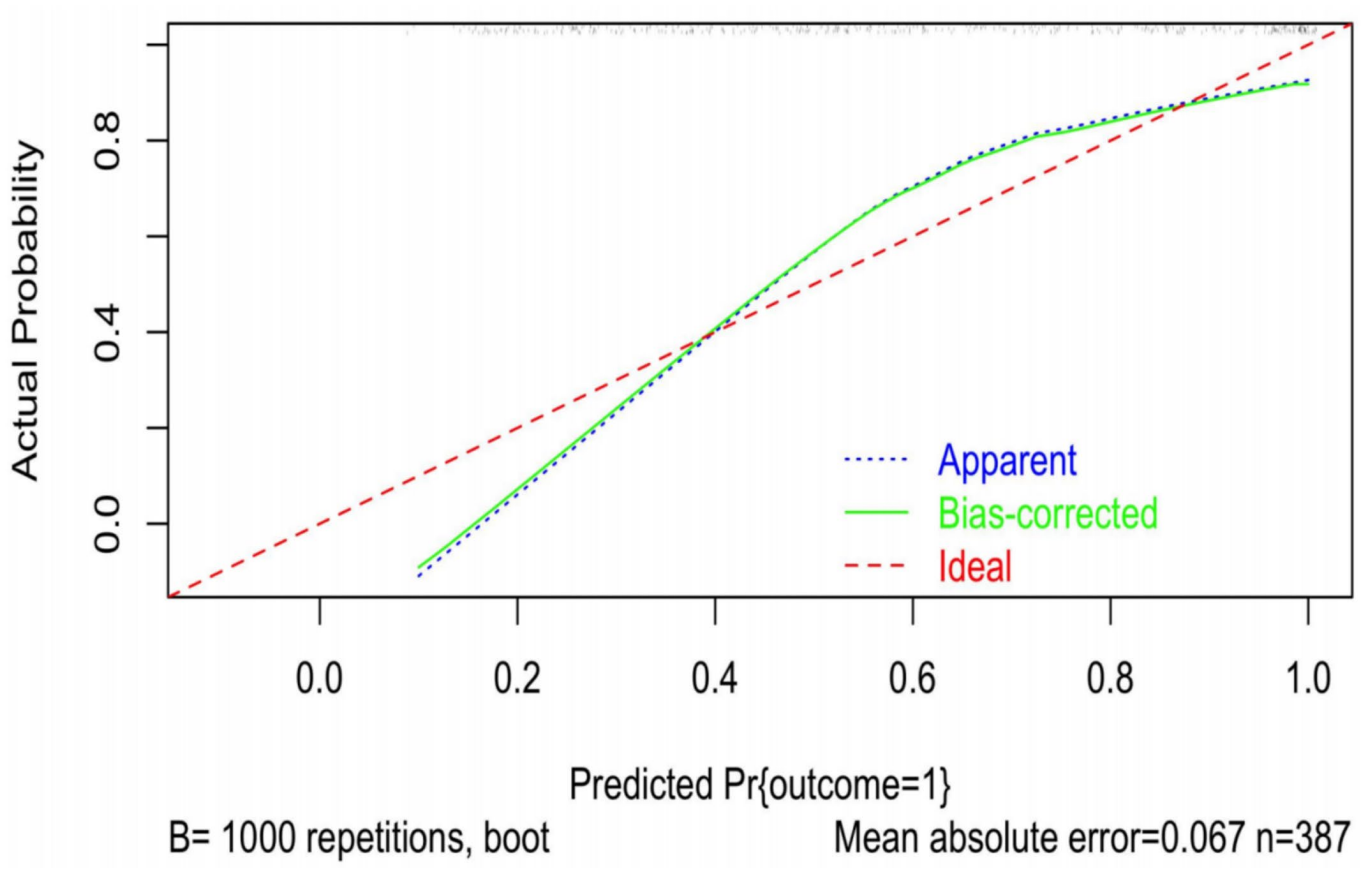
Predictive modeling effectiveness evaluation chart correction curve bootstrap method.

the optimal prediction model in this study was a multifactorial logistic regression model constructed based on the SII index, with an area under the ROC curve AUC = 0.954 (95% CI: 0.925–0.983), a sensitivity of 0.839 when the model cutoff = 0.497, a specificity of 0.755, PPV of 0.843, NPV of 0.750, and accuracy of 0.806 (95% CI: 0.763–0.844), as detailed in Figure 3. The model was analyzed for goodness-of-fit, and it was found that the model had a good ability to match between the predicted risk and the actual results (Hosmer–Lemeshow test, *X*^2^ = 4.899, *p* = 0.265 > 0.05, Figure 4).

### 3.5 Visualization of SHAP values for the importance of variables in the prediction model

**Figure 5:**
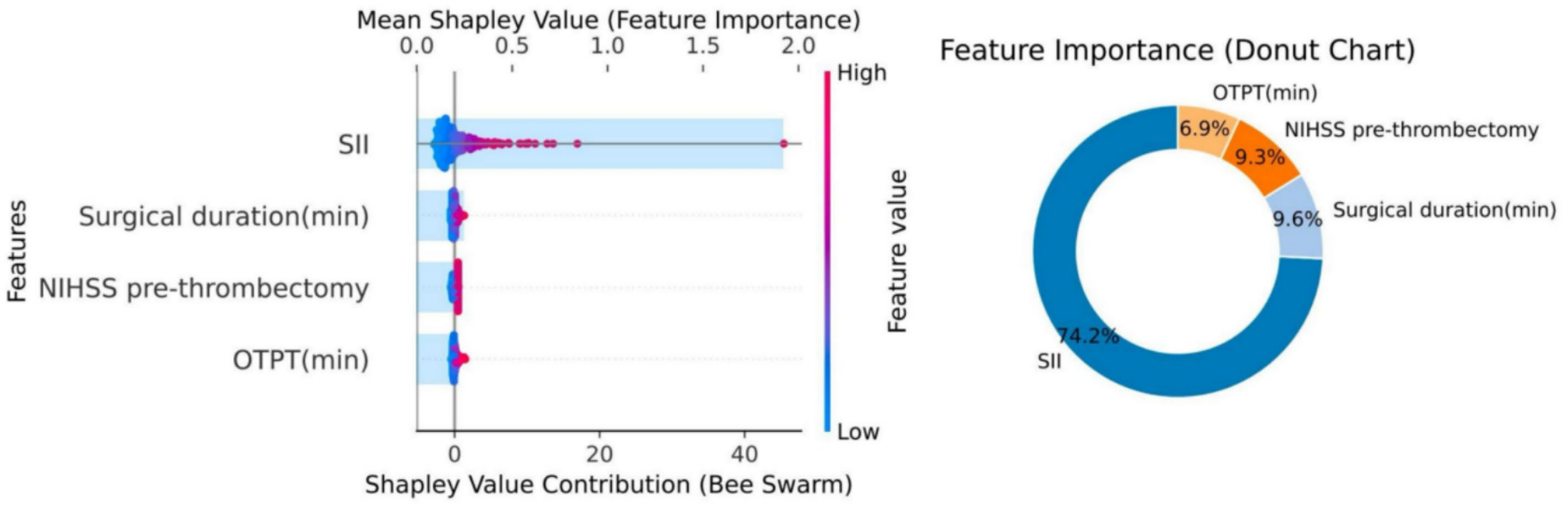
SHAP abstract map of predictive models. In the SHAP diagram, the width of the horizontal bars in the summary on the left indicates the degree of influence, suggesting a greater contribution to a wider range. The color gradient from blue (low) to red (high) reflects the magnitude of each predictor, indicating their relative importance. On the right is the percentage contribution of the different variable characteristics of the predictive model to the model.

The relative importance and impact of variables in the model in predicting postoperative functional outcome were analyzed by SHAP values, and it can be seen in Figure 5 that different variable characteristics have different impacts on prognostic prediction. In the present study, we found that SII, operative duration (min), nihss before MT, and onset-to-puncture time (min) were associated with an increased risk of poor functional outcome, and the higher the value, the higher the probability of poor functional outcome. In addition, we can find that the characteristics of different variables affect the model differently, with SII having the highest influence on the predictive model, accounting for 74.2% of the total model, followed by the length of the procedure (min) and the nihss prior to the removal of the embolus, accounting for 9.6 and 9.3% of the total model, and lastly, the time from onset of the procedure to the puncture (min), at 6.9%.

### 3.6 Results of DCA analysis

**Figure 6:**
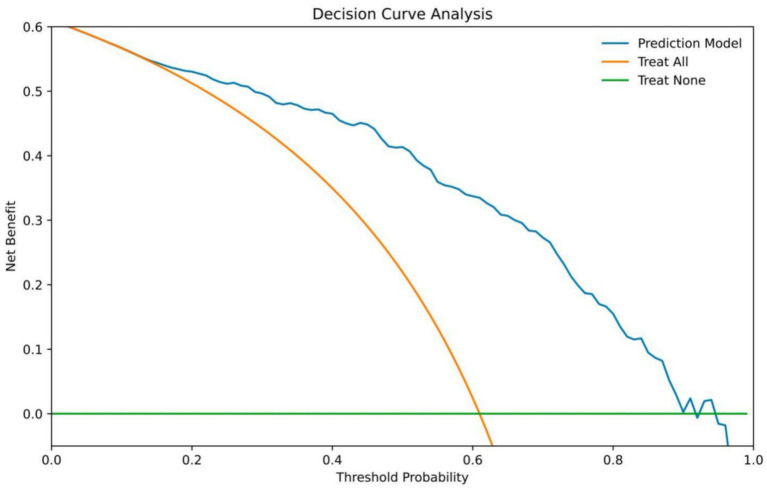
Decision curves for predictive modeling.

Figure 6 shows the decision curve analysis. For threshold probabilities (Pt) ≥ 15%, the predictive model yields a higher net benefit than the ‘treat-all’ or ‘treat-none’ strategies. For example, at Pt = 15%, the model would correctly identify approximately 15 additional poor-outcome patients per 100 screened without increasing the false-positive rate. Within the Pt range of 0.15–0.90, the model provides a consistently high net benefit for predicting poor functional outcome.

## 4 Discussion

This study demonstrates that the systemic immune-inflammation index (SII) offers a significant advantage in predicting 90-day functional outcome after mechanical thrombectomy (MT) in acute ischemic stroke (AIS) patients, with our SII-based Model 2 achieving a superior AUC of 0.863 compared to models using PLR or NLR. SHAP analysis confirmed SII as the most influential variable (74.2% contribution), underscoring its multidimensional integration of neutrophils, platelets, and lymphocytes, which captures complex inflammatory and immune imbalances more effectively than single-pathway markers like PLR (platelet activation focus) or NLR (stress/infection focus) (13, 17, 18). High SII reflects simultaneous platelet overactivation (19, 20), neutrophil infiltration and formation of neutrophil extracellular traps (NETs) (21–23), and lymphocyte depletion (24, 25), collectively exacerbating blood–brain barrier disruption, oxidative stress, and secondary brain injury (4, 26, 27), aligning with our findings of elevated SII in the poor-functional outcome group (60.98% incidence). Peripheral blood cells also exhibit rapid gene expression changes in neutrophils and monocytes post-stroke (28), further supporting the systemic inflammatory response captured by SII (4, 11, 12). Other key predictors in the model, including onset-to-puncture time, procedure duration, and baseline NIHSS, contributed 6.9, 9.6, and 9.3%, respectively, via SHAP, highlighting how procedural delays amplify ischemia–reperfusion injury and systemic inflammation (26, 29). Compared to traditional markers, SII’s multivariable-adjusted AUC (0.841) outperformed PLR (0.810) and NLR (0.818), with NRI and IDI confirming Model 2’s improvements over baselines, contrasting with prior studies on intravenous thrombolysis (9, 13, 30) by innovating MT-specific risk stratification with revascularization details. Clinically, the model’s good net benefit at a 15% DCA threshold supports early high-risk identification for optimized interventions like anti-inflammatory therapies (e.g., IL-1β inhibitors or NET inhibitors) (31, 32), with SHAP enhancing interpretability for individualized care. Our approach combines logistic regression for transparent odds ratios with SHAP for patient-specific insights, ensuring clinical adoptability and robustness (validated via 1,000 bootstrap resamples). Despite strengths such as comprehensive biomarker comparison in a sizable cohort, limitations include the single-center retrospective design, reliance on baseline SII without dynamic monitoring, and moderate sample size restricting subgroup analyses. Additionally, due to the retrospective design of this study, unmeasured confounding factors may exist. For example, specific medication use (including dosage and duration of antiplatelet agents, anticoagulants, and anti-inflammatory drugs), postoperative complications (such as infection, reperfusion injury, or pneumonia), and other potential influencing factors (such as detailed smoking history, nutritional status, and socioeconomic factors) may not have been fully incorporated into the multivariate analysis. Although we controlled for known key clinical variables (e.g., time from onset to puncture, procedure duration, and admission NIHSS) via logistic regression analysis, these unmeasured confounders may still exert some influence on the strength and interpretation of the association between SII and 90-day outcomes, potentially introducing residual bias in the findings. Therefore, future multicenter prospective studies should strengthen the collection and adjustment for these factors to further validate the robustness of this model. Future multicenter prospective studies could address these by expanding samples and incorporating advanced algorithms for non-linear associations. Furthermore, exploring postoperative SII trajectories, multimodal imaging integration (e.g., CTP/DWI), and SII-stratified RCTs for anti-inflammatory efficacy in high-risk groups, including hemorrhagic or cardioembolic strokes, would further refine.

## 5 Conclusion

This study confirms the validity of SII in the prognostic assessment after mechanical thrombectomy in acute ischemic stroke. Its integration of multidimensional inflammatory and immune information significantly improved the accuracy and clinical utility of the prediction model. Future studies need to further optimize the model design and explore precise intervention strategies under the guidance of SII to ultimately improve the long-term neurological outcome of AIS patients.

## Data Availability

The original contributions presented in the study are included in the article/supplementary material, further inquiries can be directed to the corresponding authors.
